# The impact of NKG2A and NKG2D receptors and HLA-E and MICA ligands polymorphisms on post-transplant complications after paediatric allogeneic HSCT: a single-centre experience

**DOI:** 10.3389/fgene.2023.1186123

**Published:** 2023-06-07

**Authors:** Jagoda Siemaszko, Marek Ussowicz, Blanka Rybka, Renata Ryczan-Krawczyk, Krzysztof Kałwak, Katarzyna Bogunia-Kubik

**Affiliations:** ^1^ Laboratory of Clinical Immunogenetics and Pharmacogenetics, Hirszfeld Institute of Immunology and Experimental Therapy, Polish Academy of Sciences, Wroclaw, Poland; ^2^ Department and Clinic of Paediatric Bone Marrow Transplantation, Oncology and Haematology, Wroclaw Medical University, Wroclaw, Poland

**Keywords:** HSC, NK cells, NK cell receptors, paediatric HSCT patients, MICA, HLA-E

## Abstract

**Introduction:** Natural Killer cells are the first subpopulation of lymphocytes that reconstitute after allogeneic haematopoietic stem cell transplantation (HSCT). Their activity is regulated by various receptor-ligand interactions, including stimulation of the activating NKG2D receptor by the MICA molecule, and inhibitory NKG2A receptor interacting with the HLA-E. In this study the research effort focused on the effect of selected NKG2A and NKG2D receptors and their ligands (HLA-E and MICA molecules) polymorphisms that may affect the pathomechanisms of post-transplant complications after HSCT in children.

**Methods:** One hundred donor-recipient pairs from a single paediatric transplantation centre were investigated. Altogether six single nucleotide substitutions (*NKG2A* rs7301582; *NKG2D* rs1049174, rs1154831; *HLA-E* rs1264457; *MICA* rs1051792, rs1063635) were genotyped, and the influence of polymorphisms was analysed on acute and chronic graft-versus-host disease (GvHD), cytomegalovirus (CMV) infection incidence, disease relapse and survival.

**Results:** The distribution of the evaluated polymorphisms did not differ between patients and their donors. The results showed a significant influence of *HLA-E* rs1264457 polymorphism in patients’ HLA-E*01:01 allele, which was associated with increased risk of CMV infection (*p *= 0.050), especially in children positive for CMV IgG before transplantation (*p* = 0.001). Furthermore, the effect of HLA-E*01:01 allele on CMV infections was more evident in children above the age of 7 years (*p* = 0.031). Strong tendencies (0.05 < *p* < 0.10) towards association with the risk of acute GvHD were also observed for the *NKG2A* or *MICA* polymorphisms of the recipients. In addition, *NKG2D* rs1154831 *AA* and *MICA* rs1063635 *GG* might play a protective role as they were not present in any recipient who died after transplantation.

**Conclusion:** In summary, there is emerging evidence that genotyping results of NKG2 receptors and their ligands, may have prognostic value for the outcome of paediatric allogeneic HSCT, but more extensive studies performed on larger groups of donors and transplant recipients are required to confirm these observations.

## 1 Introduction

Allogeneic haematopoietic stem cell transplantation (HSCT) is indicated in a broad range of malignant and non-malignant diseases in children (Kai and Hara, 2003; Barrett et al., 2010; Wachowiak et al., 2018). According to the 2019 European Society of Blood and Marrow Transplantation (EBMT) survey, the main indications for allogeneic HSCT in paediatric patients were acute lymphoblastic leukaemia (ALL; 24.5%), acute myeloid leukaemia (AML; 13.3%), and non-malignant disorders (49.1%) including primary immune disease and bone marrow failures, like severe aplastic anaemia (Passweg et al., 2021). It has been reported that in patients with disease-free survival 2 years post-HSCT, the probability of long-term survival reached 89% (Socié et al., 1999; Bhatia et al., 2007). Despite its beneficial effects and high survival rate, HSCT is associated with the risk of developing early and late complications (Armenian et al., 2011; Lawitschka and Peters, 2018). In addition to primary disease relapse and infections, acute and chronic graft-versus-host-disease (GvHD) are the major causes of post-transplant mortality (Vrooman et al., 2017). Based on the 11-year follow-up study, up to 60% HSCT recipients develop acute GvHD (aGvHD) and 20%–50% chronic GvHD (cGvHD). The most frequently affected organs are skin, gastrointestinal tract, and liver (Ghimire et al., 2017; Hierlmeier et al., 2018). Among post-transplant opportunistic infections, human cytomegalovirus (CMV) infection is most common and diagnosed in up to 70% of transplant recipients (Cho et al., 2019).

Orchestrated by a wide range of activating and inhibitory receptors, natural killer (NK) cells are the first donor-delivered lymphocytes regenerating after HSCT (Cooley et al., 2018). In peripheral blood, they constitute 5%–20% of lymphocytes. The NK cells act by cytokine and chemokine secretion and by the release of granulocytes, which are apoptosis-inducing proteins. In contrast to T cells, the NK-cell cytotoxicity is independent of the previous exposure to a given antigen (Vivier et al., 2008; Jelenˇci´c et al., 2017). These unique cells are divided into two major subpopulations: CD56bright and CD56dim, in which the CD56 surface antigen is being expressed or not. The CD56dim NK-cell subpopulation is characterised by strong cytolytic properties, and CD56bright are best known for cytokine secretion (Tarannum and Romee, 2021). NK cells have different features in children, especially in newborn babies. It has been proven that newborns’ NK cells have ap. 50% less cytotoxic properties, compared to adults. In such young children, the NK-cell functioning is strongly limited (e.g., they produce less IFN-γ, and they do not respond to IL-2 and IL-15 as much as mature NK cells) (Ivarsson et al., 2013; Simon et al., 2015). The immune system of children is under development for the first 7–8 years and in the first stages of life is mostly dependent on innate immune cells (Kloc et al., 2020).

The NKG2A and NKG2D receptors are members of the natural killer C-type lectin-like family recognising non-classical HLA molecules as their ligands, such as HLA-E and MHC class I polypeptide-related sequence A and B (MICA/MICB) proteins, respectively (Siemaszko et al., 2021). The NKG2A–HLA-E pathway induces a cascade of inhibitory signals resulting in an inhibition of cytotoxic activity and cytokine production by NK cells. On the other hand, the activating signal transduced by the MICA-NKG2D interaction stimulates NK cells and provides co-stimulatory signal to T-cell subsets.

**FIGURE 1:**
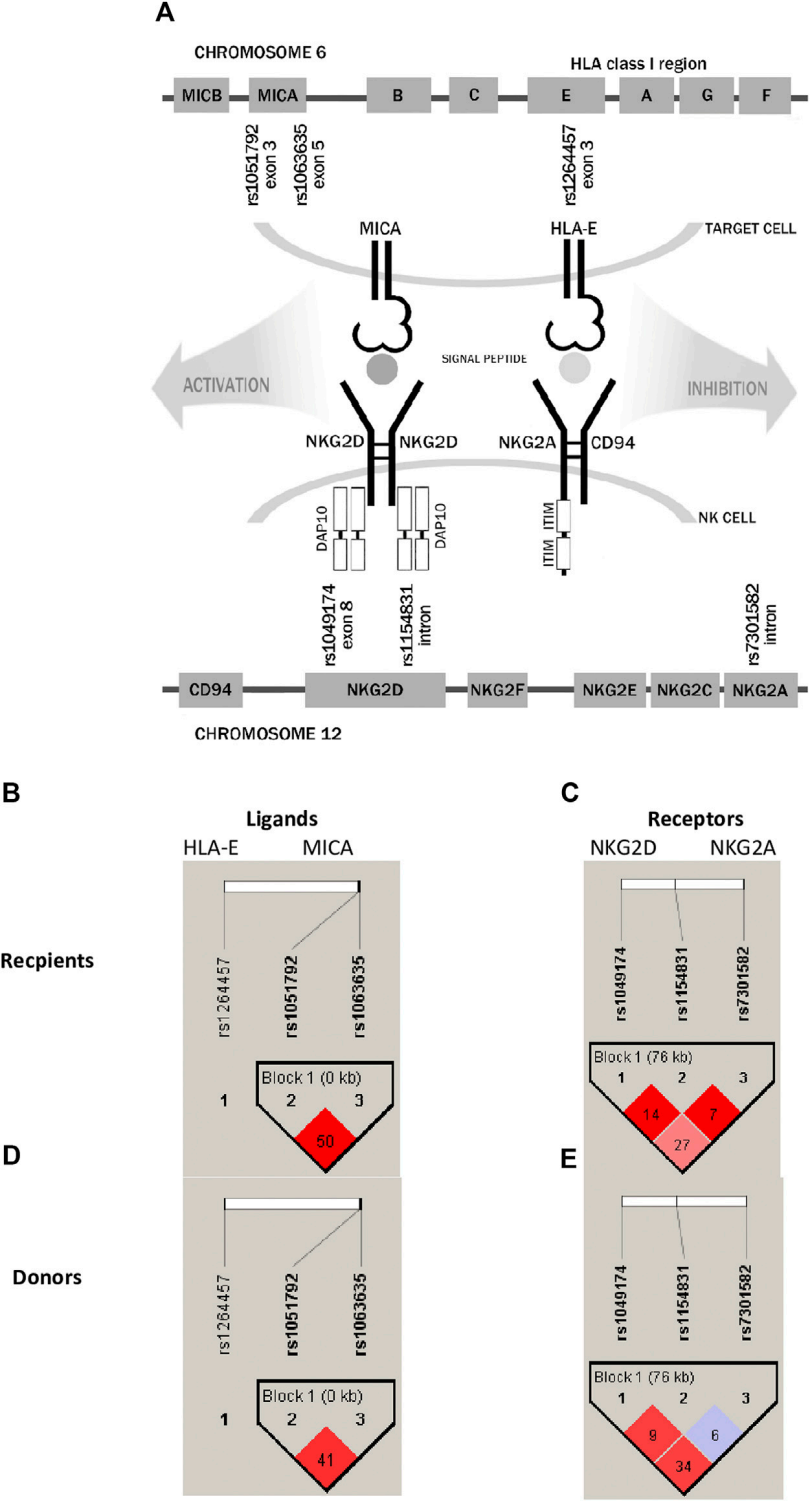
Schematic representation of analysed receptor–ligand pairs and single-nucleotide polymorphisms studied in paediatric patients and their donors. **(A)** SNP localisation. **(B,D)** Linkage disequilibrium between HLA-E and MICA ligand SNPs. **(C,E)** LD between NKG2D and NKG2A receptor SNPs. The results for recipients are shown in the upper panel, while for donors, they are shown in the lower panel. LD values as R-squared.

Genes encoding both non-classical HLA molecules, HLA-E and MICA, are located within the major histocompatibility complex on chromosome 6p21.3, while their NKG2 receptors are encoded by genes located in the natural killer gene complex (NKC) on human chromosome 12p12.3–13.2 (Figure 1A). In most populations, there are two most frequent HLA-E molecules that differ in one amino acid exchange at position 107 of the *α*-heavy-chain domain, arginine (*01:01) to glycine (*01:03), which is a result of the rs1264457 single-nucleotide polymorphism (SNP), a *T* to *C* substitution (C = *01:03/T = *01:01 Arg [T]107Gly [C]). Interestingly, these alleles affect the density of cell surface expression and peptide-binding affinity in favour of the HLA-E*01:03 variant (Braud et al., 1998; Gumá et al., 2005; van Bijnen et al., 2011; Zaguia et al., 2013). The MICA rs1051792 non-synonymous polymorphism is positioned in exon 3 and comprises a single base change (G/A) leading to substitution of Met for Val at position 129 of the alpha 2-heavy-chain domain of the MICA protein. This substitution is implicated in the functional differences of the MICA protein by modulating the strength of MICA binding to the NKG2D receptor and affecting NKG2D signalling (Isernhagen et al., 2016).

The *NKG2D/KLRK1* gene is also characterised by a functional polymorphism affecting the NK-cell activity. The rs1049174 (C>G) is a synonymous substitution positioned in the 3′ untranslated region (UTR) of the *KLRK1* gene. The rs1049174 (G>C) substitution may have an impact on NK cytotoxic activity. Molecular studies of Espinoza et al. (2016) revealed an impairment in the base pairing between miR-1245 and the regulatory region of NKG2D mRNA, transcribed from the rs1049174 *G* variant, resulting in higher NKG2D expression *in vitro* (Espinoza et al., 2016). The cytotoxic properties of the NKG2D receptor are strongly affected by its haplotypes creating two haploblocks defined as LNK1/2 and HNK1/2, which together form six different allele combinations (Hayashi et al., 2006; Espinoza et al., 2009a; Vazquez-Gonzalez et al., 2019). The LNK1 and LNK2 alleles are associated with low NK-cell cytotoxicity, while HNK1 and HNK2 are associated with high NK-cell cytotoxicity. These different haplotypes have been proven to influence the HSCT outcome (Machuldova et al., 2021a). Another relevant SNP, rs2255336 (A>G), is a non-synonymous polymorphism resulting in an Ala72Thr substitution. This SNP does not affect directly the expression of the *NKG2D*/*KLRK1* gene (Mariaselvam et al., 2017), although proteins encoded by the *KLRK1* rs2255336 variants may differ in affinity to the DAP10 adapter molecule and upon binding NKG2D ligands may transmit signals of different strengths inside activated NK cells. Among SNPs within the *NKG2A*/*KLRC1* gene, the rs7301582 polymorphism (C>T substitution in the intronic sequence) showed some clinical implications (Iwaszko et al., 2016).

There have been a number of studies suggesting some associations of polymorphisms within genes coding for NKG2 receptor ligands and the transplant outcome in adult HSCT recipients (sometimes with inconsistent results), but—to the best of our knowledge—none of them investigated paediatric patients subjected to the allogeneic HSCT procedure [reviewed in Partanen et al. (2020) and Bogunia-Kubik and Łacina (2021)]. One of the selected SNPs, the *NKG2A* rs7301582, has not been investigated in HSCT settings. However, our previous studies on patients with inflammatory arthritis receiving anti-TNF therapy documented its functional effect as showed that the presence of the *C* allele or the *CC* genotype was more frequent among patients with lack of response to anti-TNF therapy (Iwaszko et al., 2016).

In this single-centre study, we aimed to investigate the associations between polymorphisms of genes encoding two NK-cell receptors and their ligands, the non-classical HLA molecules, and the outcome of HSCT in a cohort of paediatric patients. This is the first study considering polymorphisms of two different NKG2 receptors together with their ligands in paediatric transplant donor–recipient pairs.

## 2 Materials and methods

### 2.1 Patients

A total of 100 paediatric HSCT recipients (median age 7; 41% females) together with their donors were enrolled in this study. The recipients were patients of the Department and Clinic of Paediatric Bone Marrow Transplantation, Oncology, and Haematology in Wroclaw, Poland. The study was approved by the Wroclaw Medical University Ethics Committee (identification code KB-561/2019). The summary of patient characteristics is presented in Table 1. All patients were diagnosed with haematological disorders, including haematological malignancies. The most frequent diagnosis was ALL, observed in 38% of patients. Most common post-transplant complications were aGvHD (in 63% of recipients) and CMV infection (34% of cases).

**TABLE 1:** | Paediatric patients’ characteristics,* N*=100 [%].

**Patients’s clinical data**			
**Patients’s age**		**Type of Donor**	
Median ± SD	7±5.56	MUD	78
**Patient’s sex**		MSD	19
M/F	59/41	Haploidentical	3
**Diagnosis**		**Sex match/mismatch**	
ALL	38	Match	64
AML	9	Mismatch	36
SAA	9	**Post-transplant complications**	
MDS	6	aGvHD I-IV / II-IV	63/30
Wiskott-Aldrich Syndrome	5	cGvHD	7
CML	4	CMV infection	34
JMML	4	EBV infection	22
Paroxymal Nocturnal Hemoglobinuria	3	CMV + EBV infection	7
Severe Combined Immunodeficiency	3	**CMV IgG status match/mismatch**	
Biphenotypic Acute Leukemia	2	Match	55
Fanconi Anemia	2	Mismatch	45
Hemophagocytic Lymphohistiocytosis	2	**EBV IgG status match/mismatch**	
X-linked Lymphoproliferative Disease	2	Match	43
Other	11	Mismatch	17
**Graft source**		Unknown	40
BM	17	**Outcome**	
PBSC	83	Dead	12
**Conditioning therapy**		Alive	88
MAC	18	**Relapse**	
RIC	69	Yes	11
NMA	13	No	89

ALL; acute lymphoblastic leukaemia, AML; acute myeloid leukaemia, SAA; severe aplastic anaemia, MDS; myelodysplastic syndrome, CML; chronic myeloid leukaemia, JMML; juvenile myelomonocytic leukaemia, MAC; myeloablative conditioning, RIC; reduced intensity conditioning, NMA; non-myeloablative conditioning, MUD; matched unrelated donor, MSD; matched sibling donor, EBV; Epstein–Barr Virus.

### 2.2 *NKG2A*, *NKG2D*, *MICA*, and *HLA-E* genotyping

For the polymorphism study, we used DNA samples isolated from blood leukocytes from patients and their donors, collected during standard diagnostic and follow-up procedures. Genotyping was performed for six selected SNPs located within genes encoding NKG2A and NKG2D receptors and their ligands HLA-E and MICA molecules. Genotyping for two *NKG2D*/*KLRK1* (rs1049174 and rs1154831) and two *MICA* (rs1051792 and rs1063635) and for *NKG2A*/*KLRC1* (rs7301582) and *HLA-E* (rs1264457) SNPs was performed with the use of LightSNiP assays (TibMOLBIOL, Germany), employing melting curve analyses. Determination of these polymorphisms was carried out in a LightCycler 480 II instrument (Roche Applied Science, Germany).

### 2.3 Statistical analysis

Overall survival (OS) was defined as the time from HSCT to death or the last report from patients with no events.

The association between genotyping results and clinical data was analysed using Fisher’s exact test. GraphPad Prism 9 (GraphPad Software, La Jolla, CA, United States) and RStudio (version 4.1.2, RStudio, PBC, Boston, MA) software were used for statistical calculations and visualisation of the obtained results. Survival analysis was calculated using Wilcoxon or log-rank tests. For analysis and visualisation of linkage disequilibrium (LD), Haploview software version 4.2 was used (www.broad.mit.edu/mpg/haploview, Broad Institute, United States). *p*-value at 0.05 was considered as statistically significant.

## 3 Results

### 3.1 Genotyping results—distribution of alleles and genotypes among patients and donors

Genotyping for six selected SNPs was performed for 200 individuals including 100 paediatric HSCT recipients and 100 donors. The results are summarised in Table 2. The distribution of genotypes and allelic frequencies showed no statistically significant differences between patients and their donors.

**TABLE 2:** | Results of *MICA*, *HLA-E*, *NKG2A*, and *NKG2D* genotyping, [N = %].

**Receptors**		**Recipients**	**Donors**	**Ligands**		**Recipients**	**Donors**
***NKG2D* rs1049174**				***MICA* rs1051792**			
*genotypes*	*CC*	10	5	*genotypes*	*AA*	10	9
	*CG*	44	58		*AG*	52	52
	*GG*	46	37		*GG*	38	39
*alleles*	*C*	64 (32%)	68 (34%)	*alleles*	*A*	72 (36%)	70 (35%)
	*G*	136 (68%)	132 (66%)		*G*	128 (64%)	130 (65%)
***NKG2D* rs1154831**				***MICA* rs1063635**			
*genotypes*	*AA*	5	3	*genotypes*	*AA*	22	22
	*AC*	36	35		*AG*	61	57
	*CC*	59	62		*GG*	17	21
*alleles*	*A*	46 (23%)	41 (20,5%)	*alleles*	*A*	105 (52,5%)	101 (50,5%)
	*C*	154 (77%)	159 (79,5%)		*G*	95 (47,5%)	99 (49,5%)
***NKG2A* rs7301582**				***HLA-E* rs1264457**			
*genotypes*	*CC*	64	63	*genotypes*	*CC*	16	17
	*CT*	31	35		*CT*	52	50
	*TT*	5	2		*TT*	32	33
*alleles*	*C*	159 (79,5%)	161 (80,5%)	*alleles*	*C*	84 (42%)	84 (42%)
	*T*	41 (20,5%)	39 (19,5%)		*T*	116 (58%)	116 (58%)

Alleles and genotypes of analysed genetic variants were segregated similarly in patients and their donors with marginal differences. The biggest (although not significant) differences in genotype distribution between patients and donors (approx. 10%) were observed for the *NKG2D* SNP rs1049174. The *NKG2D* SNP rs1049174 *LNK/HNK* (*CG*) heterozygosity occurred more often in donors (58%), whereas in recipients, the dominant genotype was found to be the *HNK/HNK* (*GG*) genotype (46%).

Analysis of linkage disequilibrium (LD) showed that polymorphisms of MICA molecule are in medium LD (*r* ^2^ = 0.50 in recipients and 0.41 in donors, Figures 1B, D), whereas polymorphisms for NKG2D receptor are in low LD (Figures 1C, E). The frequencies of NKG2 receptor haplotypes were similar in both groups with the most common GCC haplotype detected with frequencies of 0.410 and 0.445 in patients and donors, respectively (Table 3). Among ligands, differences in haplotypes distribution were observed. Three most common haplotypes (TGG, CGG, and TAA) occurred with different frequencies in both studied groups. In recipients, the most frequent haplotype was TGG (0.261), while in donors, it was CGG (0.251). The various frequencies of those haplotypes in patients and donors may impact the further differences in donor–recipient matching for non-classical class I HLA alleles. Indeed, there were some donor–recipient pair mismatches with respect to the HLA-E (27 pairs) and both MICA SNP (six pairs for rs1051792 and eight pairs for rs1063536) alleles. There were 27 donor–recipient pairs mismatched with respect to the classical HLA alleles: HLA-A (11 pairs), HLA-B (seven pairs), HLA-C (seven pairs), HLA-DRB1 (five pairs), and HLA-DQB1 (six pairs), mainly with HLA class I alleles.

**TABLE 3:** | Haplotype frequencies of single-nucleotide polymorphisms studied in paediatric patients and their donors. Frequencies of *HLA-E* and *MICA* SNP haplotypes are shown on the left, while *NKG2D* and *NKG2A* haplotypes are shown on the right.

**Ligands (chr. 6)**						**Receptors (chr. 12)**					
**Recipients**			**Donors**			**Recipients**			**Donors**		
HLA-E	MICA		HLA-E	MICA		NKG2D		NKG2A	NKG2D		NKG2A
rs1264457	rs1051792	rs1063635	rs1264457	rs1051792	rs1063635	rs1049174	rs1154831	rs7301582	rs1049174	rs1154831	rs7301582
1	2	3	1	2	3	1	2	3	1	2	3
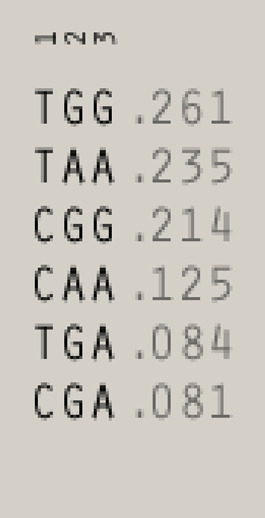			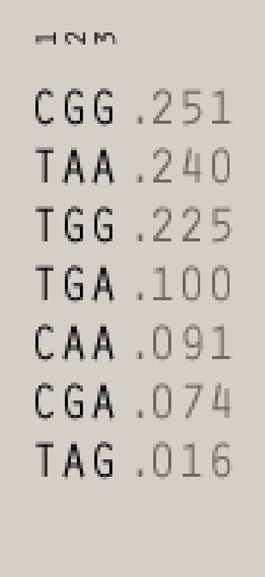			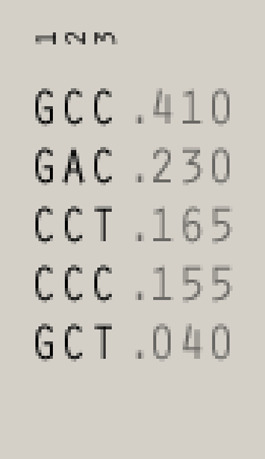			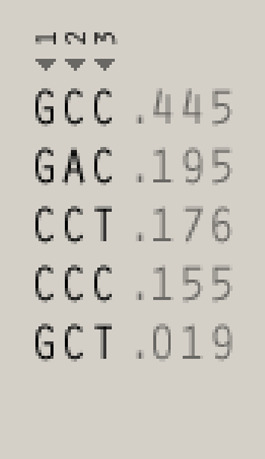		

### 3.2 Incidence of post-transplant complications—the impact of donor–recipient matching for non-classical HLA alleles

Post-transplant complications such as aGvHD may be affected by the lack of compatibility between the recipient and its donor. Both classical and non-classical HLA mismatches may increase the risk of aGvHD development. And indeed, over 70% of recipients with at least one classical HLA mismatch developed aGvHD in stages I–IV and 55% in stages II–IV (although these differences did not reach statistical significance, *p* = 0.243 and *p* = 0.218, respectively).

Interestingly, the presence of non-classical HLA mismatches seemed to additionally affect the occurrence of aGvHD. Among donor–recipient pairs matched for classical HLA alleles that were mismatched for non-classical HLA, 71.4% patients developed aGvHD as compared to 56.6% recipients matched with their donors for both classical and non-classical HLA loci. This effect was especially seen for the HLA-E mismatch (70%). Donor–recipient classical and non-classical HLA mismatch did not seem to increase the risk of other HSCT complications (cGvHD, viral infections, relapse incidence, or probability of survival).

### 3.3 Effects of particular alleles/genotypes on post-transplant complications

#### 3.3.1 aGvHD

**FIGURE 2:**
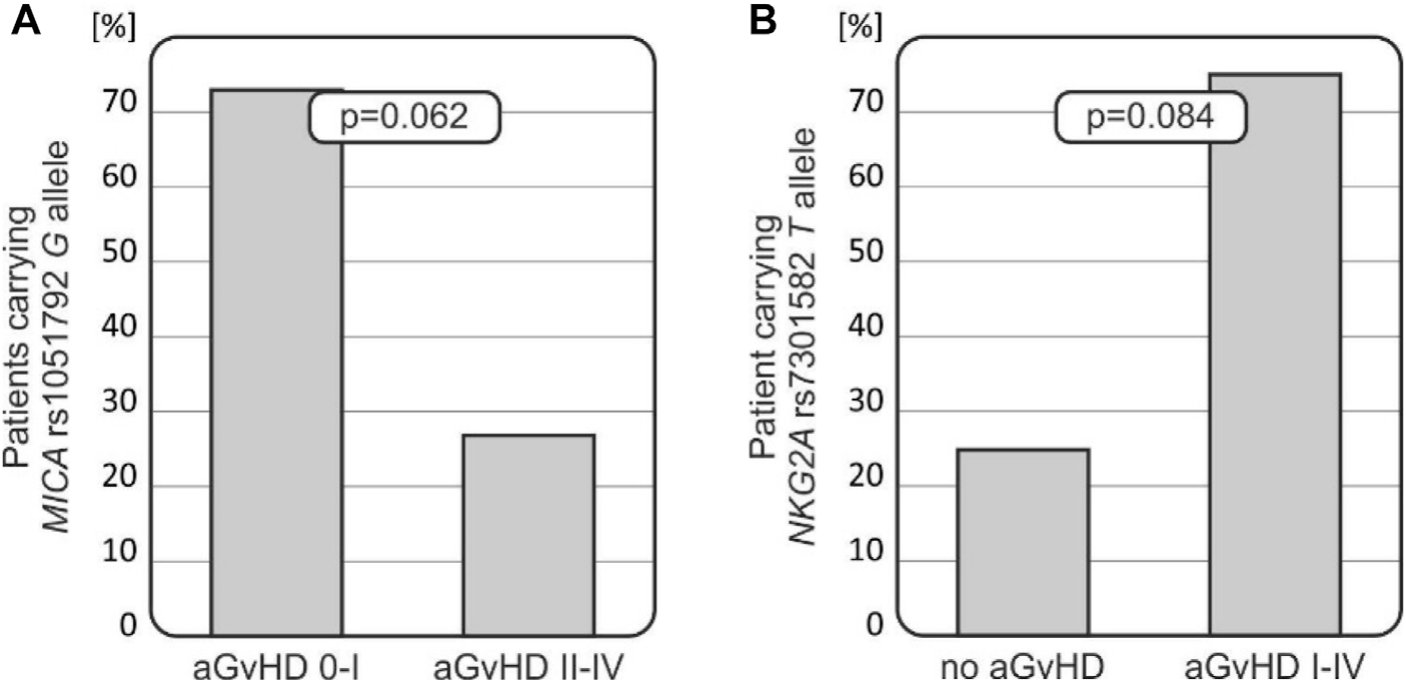
Relationships of *MICA* and *NKG2A* polymorphisms with the risk for aGvHD development. **(A)** *MICA* rs1051792 *G* allele was less frequent among recipients who developed more severe aGvHD grades II–IV. **(B)** Recipients who did not develop aGvHD carried the *NKG2A* rs7301582 *T* allele less frequently.

The presence of an aGvHD (grades I–IV) and clinically more significant (II–IV) and severe (III–IV) grades was compared in patients carrying various HLA-E, MICA, NKG2A, and NKG2D alleles and genotypes. The most noteworthy, although not significant, observations are summarised in Figure 2.

Patients carrying the MICA rs1051792 *G* (Val) allele tended to develop less-severe aGvHD (*p* = 0.062, Figure 2A). It was observed that 73.3% of recipients with *AG*/*GG* genotypes developed aGvHD grade I or did not develop this disease at all (it was observed that 66 patients without aGvHD or with aGvHD grade I were carrying the *G* allele). Among 10 recipients with MICA rs1051792 *AA* homozygosity, a majority of them developed aGvHD grades II–IV. The MICA rs1051792 *GG* homozygosity prevailed in patients without aGvHD or with stage I, compared with patients who developed stages II–IV; 29 cases (41.43%) vs. 9 cases (30%).

The frequency of the NKG2A rs7301582 *T* allele was decreased in recipients who did not develop aGvHD (25%), while this genetic variant was observed in 75% of patients who developed aGvHD of any grade (*p* = 0.084, Figure 2B).

#### 3.3.2 cGvHD

Chronic GvHD was diagnosed in seven out of 100 patients after transplantation. None of the patients with cGvHD carried the MICA rs1063635 *AA* genotype (0/7 cases). This genetic variant was present in 22 (24%) of patients without symptoms of cGvHD. The *G* allele was observed in all seven recipients with cGvHD (*p* = 0.341). As for the other analysed polymorphisms, NKG2D rs1154831 *AA* and MICA rs1051792 *AA* and NKG2A rs7301582 *TT* variants were absent in recipients who developed cGvHD.

#### 3.3.3 Viral infections

CMV infection occurred in 34 out of 100 patients after transplantation, and EBV infection was observed in 22 cases. In seven cases, patients developed both CMV and EBV infections. As expected, CMV infection was more commonly seen in patients with a positive anti-CMV IgG status before transplantation (*p* = 0.0096).

**FIGURE 3:**
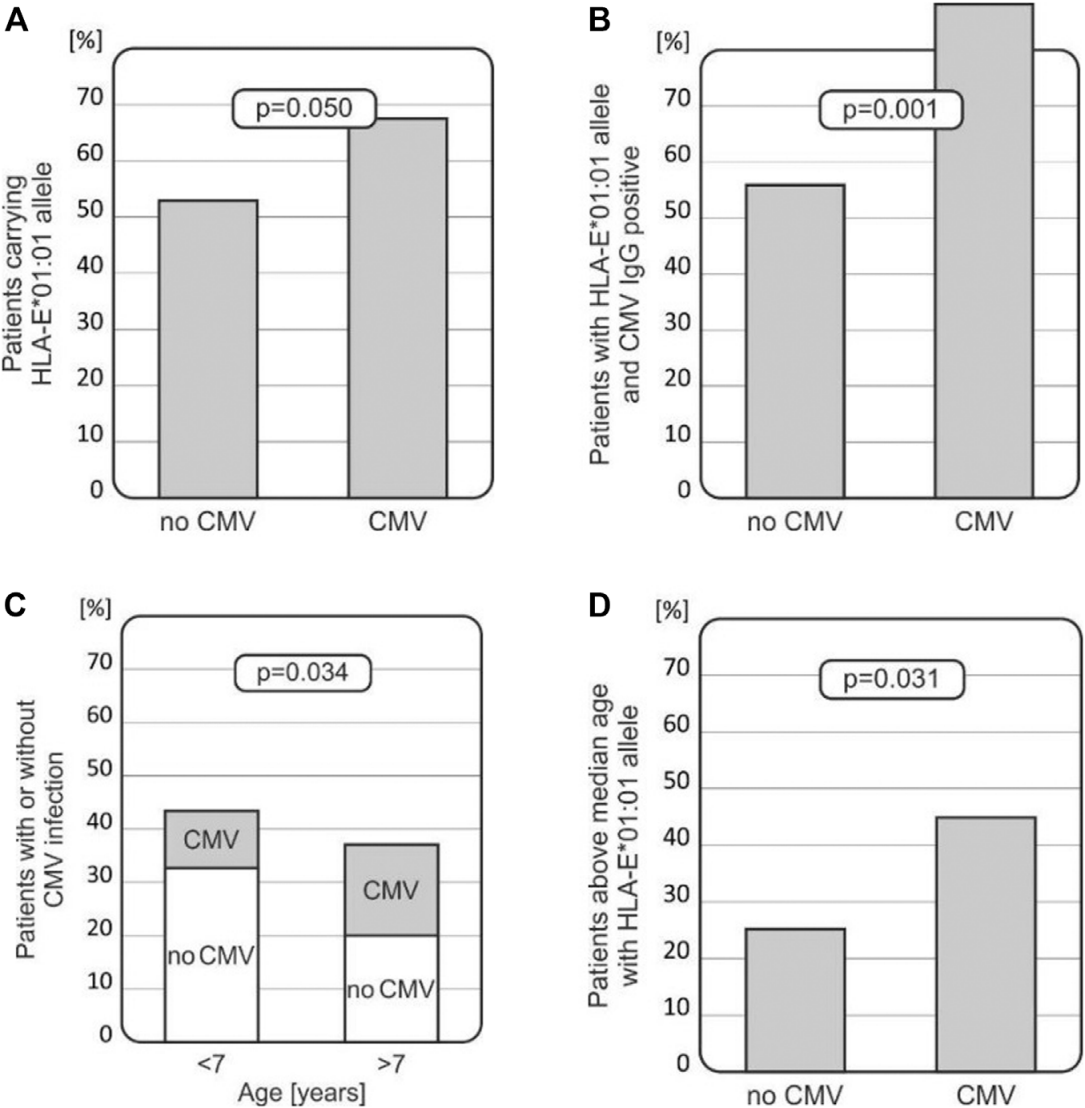
Associations between CMV infection and various clinical and genetic factors in transplant recipients. **(A)** HLA-E*01:01 (rs1264457 *T*) allele prevails in patients with CMV infection. **(B)** Recipients with the HLA-E*01:01 (rs1264457*T*) allele with positive CMV IgG status before transplantation developed CMV infection more often. **(C)** Incidence of CMV infection is higher in older children (above the median age of 7 years). **(D)** Children above 7 years of age, carrying the HLA-E*01:01 (rs1264457 *T*) allele, had an increased risk for CMV infection.

Intriguingly, among recipients who developed CMV infection after HSCT, a higher HLA-E *T* (*01:01) allele frequency was noted when compared with those without the infection (67.65% vs. 53.03%, *p* = 0.050, Figure 3A). Furthermore, recipients with the HLA-E *T* (*01:01) allele, who were positive for CMV IgG before transplantation, had higher incidence of CMV infection (*p* = 0.001, Figure 3B). Thus, the HLA*01:01 allele was found to be associated with CMV infection, especially in children positive for CMV IgG before transplantation. There was a strong association between the risk of CMV infection reactivation and the recipients’ age. Children above 7 years of age were more likely to develop CMV infection compared to the younger recipients (*p* = 0.034, Figure 3C). Finally, the presence of the HLA-E *T* (*01:01) allele increased the risk of CMV infection in this group of transplant recipients (*p* = 0.031, Figure 3D) as well.

In the case of EBV, the NKG2D rs1154831 *AA* genotype was not found in any of the patients who developed EBV infection (0/22 cases). Moreover, this genetic variant was not found in any of the patients with relapse or who died after transplantation.

#### 3.3.4 Relapse

There were no statistically significant relationships between any of the studied polymorphisms and relapse of underlying disease after transplantation.

#### 3.3.5 Survival

Among all the studied polymorphisms, those located within genes coding for the NKG2D receptor and its MICA ligand appeared to play a role with respect to the patients’ survival.

**FIGURE 4:**
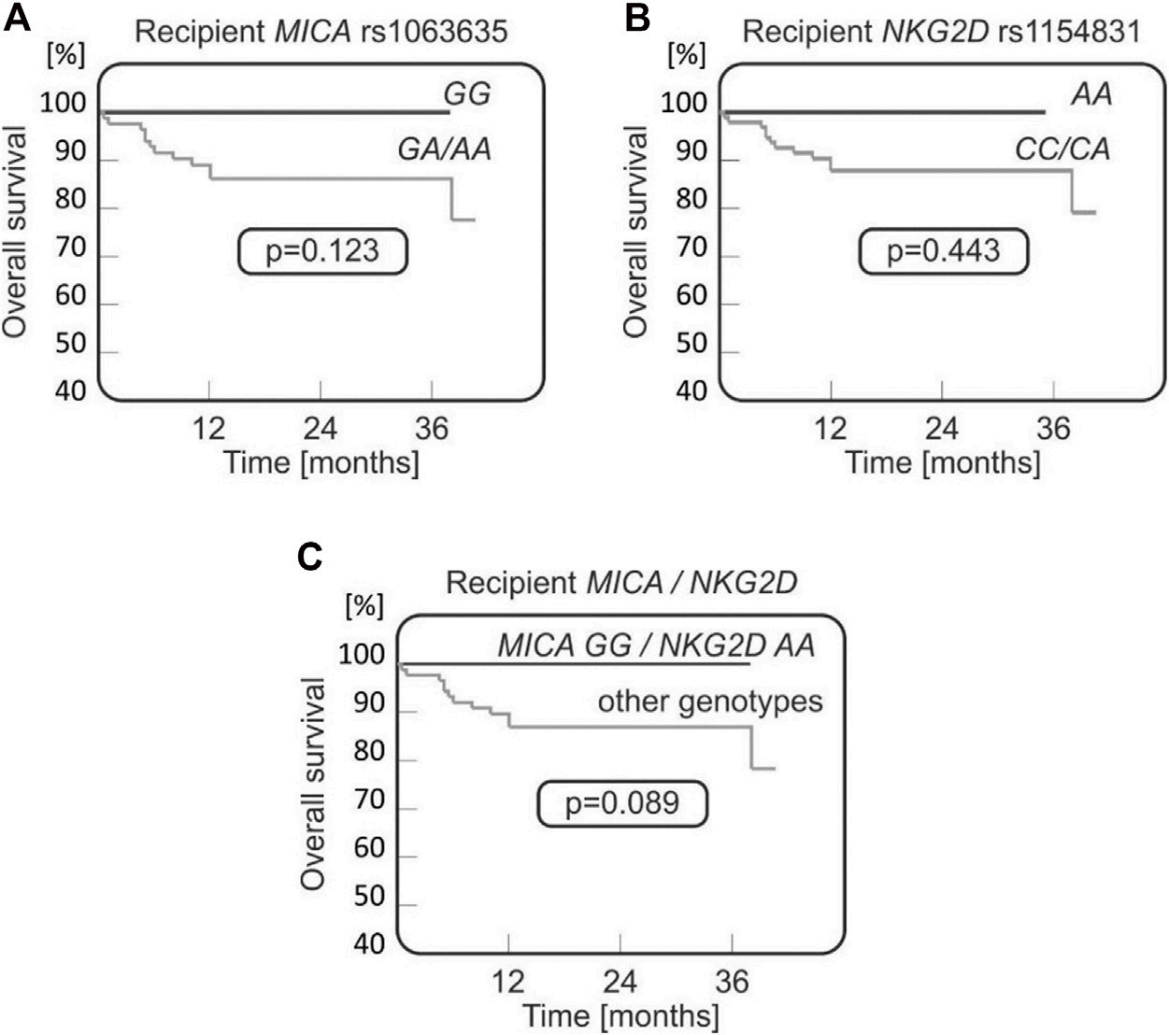
Survival curves for paediatric HSCT recipients with respect to *NKG2D* and *MICA* variants. No fatal outcomes were seen in recipients carrying *MICA* rs1063635 *GG* **(A)**, being homozygous for *NKG2D* rs1154831 *A* **(B)**, or carrying both *MICA* rs1063635 *GG and NKG2D* rs1154831 *AA* genotypes **(C)**.

It was observed that both MICA rs1063635 *GG* and NKG2D rs1154831 *AA* genotypes may have a protective effect as they were not present in any of the patients who died after transplantation (0/12 cases), while they were detected, respectively, in 19.3% and 5.7% of transplant survivors (17 and 5 recipients, Figures 4A, B). A stronger association was observed when recipients homozygous for these two polymorphisms were analysed (Figure 4C).

## 4 Discussion

NK cell activity is driven by various receptor–ligand interactions, including stimulation of the activating NKG2D receptor by the MICA protein, and binding of HLA-E to the inhibitory NKG2A receptor (Steinle et al., 2001). However, the effects of these interactions may also be dependent on the genetic variability of DNA sequence coding for receptors and their respective ligands.

There is evidence that donor–recipient incompatibility for major HLA loci constitutes a risk factor of the less-favourable transplant outcome, including the development of acute and chronic GvHD. Moreover, lack of compatibility for non-classical HLA class Ib genes was also found to increase the risk of post-transplant complications as shown for HLA-E incompatibility and increased aGvHD risk (Bogunia-Kubik et al., 2009; Bogunia-Kubik et al., 2010; Bogunia-Kubik et al., 2011) or increased incidence of acute and chronic GvHD and worse survival after MICA mismatched HSCT in the 10/10 HLA-matched donor–recipient pairs (Fuerst et al., 2016). Similar observations regarding MICA incompatibility were documented by Parmar et al. (2009), Askar et al. (2014), Carapito et al. (2016), and Askar et al. (2017). More recently, an increased risk of acute and chronic GvHD and an increased risk of CMV reactivation were also reported after MICB mismatched transplants (Carapito et al., 2020). All these studies involved adult transplant recipients, and similar effects were seen in our paediatric cohort of HSCT patients. We observed a higher incidence of aGvHD after HLA incompatible transplants and after transplantation with donor mismatched for non-classical HLA loci, especially for HLA-E. However, these relationships did not reach statistical significance, most probably due to the low number of donor–recipient pairs tested.

Analyses of the effect of a particular genetic variant on the transplant outcome showed some interesting results regarding NKG2A and MICA variants. In contrast to the NKG2A rs7301582 *T* allele, the presence of the MICA rs1051792 *G* (129Val) allele in the recipient seems to decrease the risk for aGvHD (Figure 2). Thus, the presence of the MICA rs1051792 *C* (Met129) allele in the recipient may have an unfavourable effect on the transplant outcome and facilitate aGvHD development after paediatric HSCT. This is also in line with the observations of Isernhagen et al. (2015) and Boukouaci et al. (2009) who reported that homozygotes for MICA-129Met alleles carry a higher risk of relapse due to the exhaustion effect. Isernhagen et al. also found that MICA-129Met clones release more soluble MICA and that the patients with sMICA >80 pg/mL after transplantation have a higher risk of developing cGvHD (Table 4) (Boukouaci et al., 2009). The single methionine (Met) to valine (Val) amino acid substitution at position 129 located on the α2-heavy-chain domain of the MICA encoding gene affects the strength of NKG2D binding. The MICA-129 Met allele binds the receptor with higher affinity, and the MICA-129 Val allele weakens the binding (Steinle et al., 2001). This feature increased the interest in studying the impact of this polymorphism on clinical outcome in various diseases. Studies on transplant recipients led to some conclusions regarding the MICA polymorphism and its impact on HSCT outcome. Patients carrying the Val allele have a higher risk of cGVHD (Boukouaci et al., 2009), while the Met allele is proven to be associated with a better OS (Isernhagen et al., 2015) and lower risk of transplant-related mortality, when the donor carries at least one Met allele (Martin et al., 2020). As described earlier, the Met variant occurred more frequently in recipients with aGvHD. Furthermore, there is evidence which indicates that intensive and rapid NK-cell activation, associated with the Met allele, promotes aGvHD development (Fuerst et al., 2016). The other investigated MICA polymorphism, rs1063630, was proved to affect the OS in HSCT recipients. The donor MICA-14Gly allele was characterised as the risk-modifying factor as it was associated with an increased OS in the recipient group (Machuldova et al., 2021b). A MICA-129 mismatch between the donor and the patient was described to increase the risk of aGvHD (Parmar et al., 2009; Fuerst et al., 2016; Carapito et al., 2020).

**TABLE 4:** | Reported clinical significance of NKG2A and NKG2D receptors and their two ligands, HLA-E and MICA molecules, in HSCT.

Polymorphism	Effect	Population studied	Reference
HLA-E rs1264457 C/T (*01:03/*01:01)	HLA-E*01:03 might support transplant tolerance via the activation of HLA-specific regulatory T cells	**-**	Wieten et al. (2014)
	Recipient *01:03 homozygosity predispose to higher overall and disease-free survival and a lower transplant-related mortality	83 HLA-matched D/R pairs	Danzer et al. (2009)
	Beneficial association of HLA-E*01:03 with lower risk of GvHD and TRM	187 HLA-identical sibling D/R pairs 124 unrelated (D)/(R) pairs	Tamouza et al. (2006) and Ludajic et al. (2009)
	Reduced cumulative incidence of relapse at 2 years was found with HLA-E*01:03 allele compared to HLA-E*01:01/01:01 genotype	88 recipients transplanted from their HLA-identical siblings	Mossallam et al. (2015)
	Better overall survival in recipients homozygous for HLA-E*01:03 Increased risk of acute GvHD after HLA-E mismatched transplants	100 allogeneic (D)/(R) pairs	Bogunia-Kubik et al. (2009), Bogunia-Kubik et al. (2010) and Bogunia-Kubik et al. (2011)
	HLA-E*01:01 homozygosity associated with increased risk of post-transplant CMV infection	100 paediatric recipients with donors	Present study
**MICA rs1051792 C/G (Met129Val)**	MICA-129 mismatch was associated with higher non-relapse mortality, higher rates of aGvHD	2 172 unrelated D/R pairs	Fuerst et al. (2016)
	Recipient homozygous for MICA-129Met alleles carriers are at higher risk to experience relapse due to the exhaustion effect	452 D/R pairs	Isernhagen et al. (2015)
		211 HLA-identical sibling D/R pairs	Boukouaci et al. (2009)
	MICA-129 Val/Val genotype and elevated sMICA serum levels after HSCT were associated with the incidence of cGVHD The presence of MICA Abs before transplantation conferred protection against cGVHD	211 HLA-identical sibling pairs and in a subset of 116 recipients	Boukouaci et al. (2009)
	Recipient *G* (Val) allele may predispose to less severe acute GvHD	100 paediatric recipients ad their donors	Present study
**NKG2D rs1049174 C/G (HNK1/LNK1)**	Donor HNK1 haplotype significantly reduced transplant-related mortality and better overall survival	145 recipients with hematologic malignancies and their unrelated donors (unrelated donors of HLA-matched myeloablative bone marrow transplants)	Espinoza et al. (2009b)

In our study, the observed distribution of MICA-129 genotypes Met/Met, Met/Val, and Val/Val was comparable in recipients and donors. Neither MICA-129 polymorphism nor MICA mismatches were associated with HCT outcomes at a level that reached the predetermined level of statistical significance. This registry analysis in patients with ALL, AML, and MDS was discordant with prior studies of patients with variable diagnoses (Askar et al., 2017). Interestingly, this unfavourable effect of the MICA rs1051792 *C* (Met129) allele we also observed in patients suffering from rheumatic inflammatory diseases with respect to the increased disease susceptibility and less-effective treatment outcome (Sokolik et al., 2014; Iwaszko et al., 2020, Wielinska et al., personal communication). These previous studies also documented the association of the MICA rs1051792 *GG* genotype with higher serum levels of the MICA protein. The results, coming from both previous and our present studies, might imply that higher expression of the MICA ligand and a proper activity of NK cells may be required for pathomechanism of GvHD. This hypothesis needs to be elucidated in further studies.

In addition, it is likely that the NKG2A and MICA polymorphisms tend to associate with the risk of aGvHD, although the results did not reach statistical significance (0.05 < *p*< 0.10) in the recipient group. For cGvHD, disease relapse, or patient survival, no significant associations were detected. Furthermore, we observed relationships between MICA rs1063635 and NKG2D rs1154831 SNPs and the probability of OS (Figure 4) with a favourable effect of the MICA rs1063635 *GG* and NKG2D rs1154831 *AA* homozygous genotypes, especially in recipient MICA rs1063635 *GG* and donor NKG2D rs1154831 *AA* constellation. The rs1154831 (NKG2D) *AA* and rs1063635 (MICA) *GG* genotypes were not present in any of the patients who died after transplantation. There are only two previous studies on NKG2D rs1154831, both considering the role of NKG2D receptor polymorphisms in rheumatoid arthritis (RA) (Iwaszko et al., 2018; Wielińska et al., 2021). One of them reported that Caucasian (Greek) patients who are *CC* homozygotes had a lower disease activity score in RA (Wielińska et al., 2021). This supports our finding that the *CC* homozygosity may have a protective effect.

As for the NKG2A rs7301582 polymorphism, the *T* allele was previously confirmed to have a protective effect as it is associated with a better response to anti-TNF therapy in RA patients (Iwaszko et al., 2016). Our results showed that the NKG2A rs7301582 *TT* variant was absent in recipients who developed cGvHD and the *T* allele occurred in just 1/7 (14.29%, *p* = 0.263) cases with cGvHD. On the other hand, the *T* allele was also observed in 75% of patients who developed the acute form of GvHD.

As expected, CMV infection after transplantation was more commonly observed in children with positive anti-CMV IgG status before transplantation. However, CMV infection was more frequent in patients carrying the HLA-E*01:01 allele (rs1264457 *T* variant) (Figure 3). This association was even more significant in patients tested positive for anti-CMV IgG before transplantation (Figure 3B). The impact of the HLA-E*01:01 allele was also seen when the recipient’s age was considered (Figures 3C, D). These findings are in line with the results coming from other studies in adult transplant recipients that documented the unfavourable role of HLA-E*01:01 (summarised in Table 4).

Interestingly, the HLA-E*01:01/E*01:01 genotype seems to be strongly associated not only with viral but also with bacterial infections. As shown by Tamouza et al., the donor HLA-E*01:01 homozygosity increased the risk of bacterial infection in BMT recipients. Additionally, it was also considered a risk factor for higher transplant-related mortality (TRM) (Tamouza et al., 2005). Here, we observed that the *TT* genotype was absent in paediatric recipients suffering from aGvHD grades III–IV, while it was detected in 35.16% of patients without or with less-severe aGvHD grades I–II (*p* = 0.054), which indicates that the *TT* homozygous genotype may have a protective effect. The HLA-E rs1264457 *TT* genotype was also associated with a better response to anti-TNF treatment in patients with RA. This genetic variant was less frequently observed in healthy controls compared to RA patients (Iwaszko et al., 2015). Furthermore, Wieten et al. (2014) suggested that HLA-E*01:03 might promote transplant tolerance via the activation of HLA-specific regulatory T cells.

In conclusion, to the best of our knowledge, this is the first study describing the distribution of polymorphisms of two different NKG2 receptors together with their ligands in a paediatric cohort of HSCT recipients and their donors. We are aware that the low number of individuals studied is the main limitation of the present study. Including a higher number donor–recipient pairs would undoubtedly increase the reliability of our single-centre study. More extended analyses including a higher number of patients and their donors would confirm the significance of our preliminary observations, suggesting that polymorphisms within genes coding for NKG2 receptors and their ligands affect the development of post-transplant complications in paediatric HSCT. Nevertheless, our results strongly support the necessity of extended donor–recipient genotyping for non-classical HLA loci (MICA and HLA-E) and further for the genes coding for receptors of these molecules.

## Data Availability

The datasets presented in this study can be found in online repositories. The data can be found at: https://cloud.hirszfeld.pl/index.php/s/F0Doz8zvEhceehW. Further inquiries can be directed to the corresponding author.
